# Affective response to exercise and physical activity engagement: preliminary results

**DOI:** 10.1002/alz.091456

**Published:** 2025-01-09

**Authors:** Marta Stojanovic, John C. Morris, Beau Ances, Denise M Head

**Affiliations:** ^1^ Washington University in St. Louis, St. Louis, MO USA; ^2^ Knight Alzheimer Disease Research Center, St. Louis, MO USA; ^3^ Washington University in St. Louis, School of Medicine, St. Louis, MO USA; ^4^ Washington University in St. Louis School of Medicine, St. Louis, MO USA

## Abstract

**Background:**

Due to multiple benefits of sustained exercise and physical activity, it is important to understand factors that can facilitate regular engagement in physical activity in later adulthood. Prior work found that a more positive affective response to acute exercise was associated with greater physical activity levels in younger adults. However, this determinant has been understudied in older adults and individuals at risk of Alzheimer disease (AD). Hence, the first goal of the study was to examine whether affective response to acute exercise predicted physical activity levels in middle‐aged and older adults. In addition, depression has been linked to reduced physical activity levels and might impact the role of affective response. Furthermore, there is often a decline in physical activity engagement with accumulation of AD pathology. Hence, the second goal was to examine whether depressive symptoms and AD genetic risk moderate the association between affective response to exercise and physical activity levels.

**Method:**

Cognitively normal individuals from the Knight ADRC Adult Children Study (n = 65; age range 44‐85 years) completed an acute bout of moderate‐intensity exercise. Affect was measured at baseline, immediately after, 15 minutes, and 30 minutes post‐exercise. Participants also completed one week of actigraphy, which provided an objective estimate of their physical activity levels. Finally, the 15‐item Geriatric Depression Scale (n = 49) and APOE phenotyping (n = 61) were obtained from a subset of individuals.

**Result:**

Greater increase in high arousal positive affect following the acute exercise was associated with higher levels of moderate‐to‐vigorous physical activity (p = 0.041). There was no moderation by depressive symptoms (p = 0.288) or APOE genotype (p = 0.962).

**Conclusion:**

These results suggest that a positive affective response to moderate‐intensity exercise might be predictive of physical activity levels across later adulthood. However, depressive symptoms and genetic risk for AD might be limited as moderators of this association. Future studies should use a larger sample size and consider additional measures of AD pathology as potential moderators.

